# A New Performance Metric to Estimate the Risk of Exposure to Infection in a Health Care Setting: Descriptive Study

**DOI:** 10.2196/32384

**Published:** 2022-02-02

**Authors:** Kimia Hadian, Geoff Fernie, Atena Roshan Fekr

**Affiliations:** 1 The KITE Research Institute Toronto Rehabilitation Institute University Health Network Toronto, ON Canada; 2 Institute of Biomedical Engineering University of Toronto Toronto, ON Canada; 3 Department of Surgery University of Toronto Toronto, ON Canada

**Keywords:** hand hygiene, health care-acquired, infection control, compliance, electronic monitoring, exposure, risk, hygiene, monitoring, surveillance, performance, metric, method, estimate, predict, development

## Abstract

**Background:**

Despite several measures to monitor and improve hand hygiene (HH) in health care settings, health care-acquired infections (HAIs) remain prevalent. The measures used to calculate HH performance are not able to fully benefit from the high-resolution data collected using electronic monitoring systems.

**Objective:**

This study proposes a novel parameter for quantifying the HAI exposure risk of individual patients by considering temporal and spatial features of health care workers’ HH adherence.

**Methods:**

Patient exposure risk is calculated as a function of the number of consecutive missed HH opportunities, the number of unique rooms visited by the health care professional, and the time duration that the health care professional spends inside and outside the patient’s room without performing HH. The patient exposure risk is compared to the entrance compliance rate (ECR) defined as the ratio of the number of HH actions performed at a room entrance to the total number of entrances into the room. The compliance rate is conventionally used to measure HH performance. The ECR and the patient exposure risk are analyzed using the data collected from an inpatient nursing unit for 12 weeks.

**Results:**

The analysis of data collected from 59 nurses and more than 25,600 records at a musculoskeletal rehabilitation unit at the Toronto Rehabilitation Institute, KITE, showed that there is no strong linear relation between the ECR and patient exposure risk (*r*=0.7, *P*<.001). Since the ECR is calculated based on the number of missed HH actions upon room entrance, this parameter is already included in the patient exposure risk. Therefore, there might be scenarios that these 2 parameters are correlated; however, in several cases, the ECR contrasted with the reported patient exposure risk. Generally, the patients in rooms with a significantly high ECR can be potentially exposed to a considerable risk of infection. By contrast, small ECRs do not necessarily result in a high patient exposure risk. The results clearly explained the important role of the factors incorporated in patient exposure risk for quantifying the risk of infection for the patients.

**Conclusions:**

Patient exposure risk might provide a more reliable estimation of the risk of developing HAIs compared to ECR by considering both the temporal and spatial aspects of HH records.

## Introduction

Health care-acquired infections (HAIs) occur during the process of care in hospitals or health care centers and were not present at the time of patient admission. HAIs cause more than 99,000 deaths and account for an additional cost of US $28-$45 billion annually in the United States [1,2]. In Canada, 48,653 cases of HAI were reported between 2017 and 2018 [3]. A study conducted in 2017 indicated that the prevalence of HAIs in Canada was 7.9% [4]. The hands of health care workers (HCWs) play an important role in spreading the pathogens responsible for HAIs in health care settings [5]. Studies have shown that hand hygiene (HH) is one of the most effective ways to reduce infection transmission in health care settings, and both alcohol-based hand sanitizers and soaps are effective in disrupting the chain of infection transmission (including viruses such as SARS-CoV-2) [5-9]. Currently, the growing number of COVID-19 cases highlights the importance of HH in infection prevention [10].

Several organizations including the World Health Organization and the Ministry of Health and Long-Term Care (MOHLTC) in Ontario, Canada have provided guidelines and recommendations for HH practices in health care environments [11,12]. According to MOHLTC’s standard precautions, HCWs should clean their hands in four moments or opportunities: (1) before initial patient or patient environment contact; (2) before aseptic procedures; (3) after body fluid exposure risk; and (4) after patient or patient environment contact [12]. Most types of HAIs can be avoided by complying to the standard HH protocols [13]. HCWs’ adherence to recommended HH procedures varies from 5% to 89% with an overall average of as low as 38.7% [13]. Identifying the poor performers as well as the population at greater risk of infection are the two key elements to understanding the underlying reasons for low compliance and directing efforts to prevent the spread of infection.

Detection of HH actions by a trained observer is currently the gold standard in HH monitoring; however, this method is not only expensive but also suffers from inadequate staffing, delayed data feedback, and an overestimate of performance between 200% and 300% [14,15]. With the recent advances in electronic monitoring systems (EMS), it is possible to track individual HCW’s HH actions automatically [16-21]. These systems provide additional information such as the length of time spent in each room, consecutive missed HH opportunities, and the number and time of entrances into and exits from each zone. This information allows us to augment existing measurements of HH performance by extending the focus from single moments to continuous time-dependent and space-dependent features in HH records. The compliance rate, which focuses on the adherence of HCWs to HH protocols, is commonly used to estimate each individual’s HH performance [5]. However, this parameter masks the temporal and spatial aspects of HH involved in infection transmission. In this study, a novel HH performance metric is proposed to quantify the individual patient’s exposure risk to infection. This parameter, called patient exposure risk, changes the focus from improving HCWs’ compliance rate to reducing the patient’s risk of infection. Different examples are discussed to show the likely ability of the new proposed metric compared to the traditional compliance rate in estimating the infection risk for patients.

## Methods

### Overview

In this paper, a new performance metric, patient exposure risk, is introduced to quantify the risk of exposure to an infection for each individual patient at a health care environment. This parameter, which is estimated for each patient’s room during 24 hours, is compared with the entrance compliance rate (ECR), a localized version of the conventional compliance rate. ECR is defined as the ratio of the number of times HH actions were performed by the HCWs upon entering a specific room to the total number of times HH action was required. Therefore, the higher the ECR, the better the performance of HH for that specific room. In other words, an ECR of 100% shows that all HCWs who entered and exited the patient’s room washed their hands.

Conventional compliance rate considers HH opportunities as isolated binary events. However, the risk of infection transmission to the patient is a multifactorial concept depending on the events prior to entering the patient’s room as well as the events happening inside the room. The risk of developing an infection in a patient depends on the number of microorganisms on the HCW’s hand, the type of microorganism, the duration of exposure, the activity performed by the HCW, and host susceptibility [22]. The new proposed patient exposure risk estimates the risk of exposure to an infection by continuously monitoring five major factors that are responsible in the chain of infection transmission but were not included in the current compliance measure. These factors include the number of consecutive missed HH opportunities, the number of unique rooms visited by the HCWs with missed HH opportunities, the length of time spent inside each patient room without performing HH, the length of time spent outside of the patient room without performing HH, and the risk factor associated with the type of pathogens present in each specific room.

Analyzing the data collected from 59 nurses at a musculoskeletal rehabilitation unit at the Toronto Rehabilitation Institute, KITE, highlighted the important role of the factors incorporated in patient exposure risk estimation. These data were collected from 10 single rooms and 10 double rooms in three 30-day trials from 2016 to 2017 using the Buddy Badge (Hygienic Echo Inc) EMS [23]. The Buddy Badge EMS provides information such as the number of consecutive missed HH opportunities, the HCWs’ location, and the time each HCW spent inside or outside of each patient’s room [24]. After cleaning the data, 85 days were considered in our analysis. Python 3.6 (Python Software Foundation) and the Spearman rank-order correlation were used for the analysis. The Spearman correlation measures the monotonic relationship between 2 variables (ie, if 1 variable increases or decreases, the other should also increase or decrease to produce a high correlation coefficient). The values in each signal will be assigned a rank variable with respect to each other. The Spearman correlation coefficient is defined as the Pearson correlation coefficient between the ranked values and is computed as:




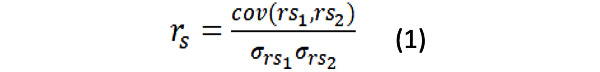




where *r_s_* denotes the Spearman correlation coefficient, *cov(rs_1_,rs_2_)* is the covariance of the rank variables, and *σ_rs_1__* and *σ_rs_2__* are the standard deviation of the rank variables [25].

### Preliminary Analysis of the Contributing Factors to the Patient Exposure Risk

**Figure 1:**
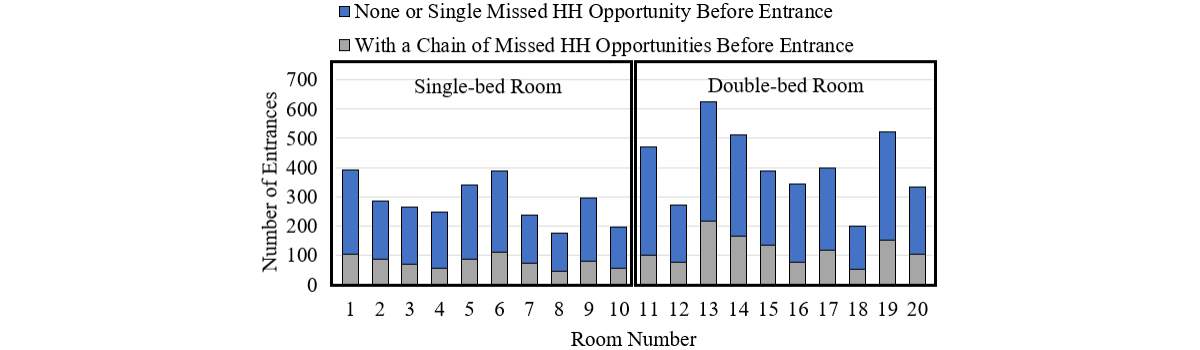
Number of entrances with and without a chain of missed hand hygiene opportunities prior to the entrance over 85 days. HH: hand hygiene.

The overall risk of infection lies in the cumulative high frequency of hands touching surfaces and patients instead of individual HH indications [22]. The authors in [23] showed that more than 80% of missed HH opportunities occur as a part of a chain with a median length of 4 and an IQR of 8, instead of a single isolated event. In addition, the results of our analysis showed that 28% (1954/6887) of the nurses entering a specific room without washing hands had a chain of missed HH opportunities (Figure 1).

It was also observed that 40% (2774/6887) of missed HH chains are made up of consecutive visits to the same room (eg, when the nurses exit the room to pick up clean supplies from the clean utility room or dispose soiled linen in the soiled utility room). Table 1 indicates the number of unique rooms visited by the nurses without handwashing before entering a specific room.

**Table 1:** Histogram of the number of unique rooms visited in the chain of missed hand hygiene opportunities over 85 days.

Number of unique rooms visited	Total number of unique rooms visited (%)
1	65.82
2	21.48
3	5.71
4	2.51
5	1.47
6	0.94
7	0.68
8	0.42
9	0.29
10	0.26
11	0.20
12	0.07
13	0.12
14	0.03

This analysis shows that in more than 34% (2354/6887) of the entrances in which HCWs did not perform HH, they had failed to perform handwashing actions in 2 or more previous visits to other rooms as well. In other words, in about 34% of entrances, the patient may have been exposed to the risk of infection from 2 or more types of infectious agents acquired in previously visited rooms.

**Figure 2:**
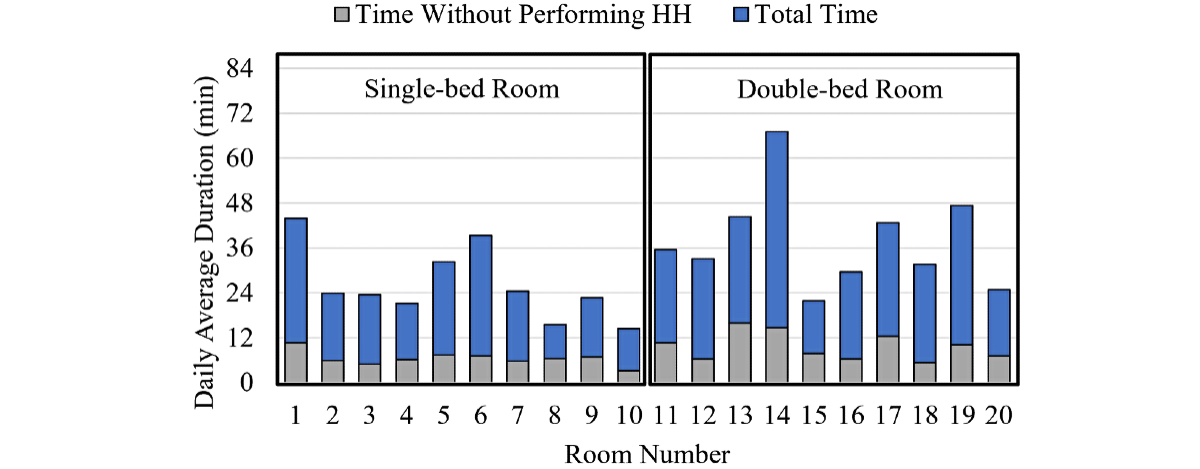
The average duration and unwashed duration spent in each room during 24 hours over 85 days; HH: hand hygiene; min: minutes.

Another important factor responsible in the transmission risk is the length of time spent in a patient room without a handwashing action. Bacterial hand contamination increases linearly over time [26]. Thus, the more time the nurse spends in each room, the more likely the patient will become infected if HH action is missed. Figure 2 depicts the average daily time duration spent in each room without HH action for the entire data set. For instance, considering room #1, the nurses spent totally about 908 minutes (~15 hours) inside this room without washing their hands upon entry, during 85 days. Therefore, on average, the nurses spent about 11 minutes during 24 hours in room #1 without washing their hands. This means that, on average, the patient in this room was exposed to risk of infection transmission from the HCWs’ hands for 11 minutes in a single day.

**Figure 3:**
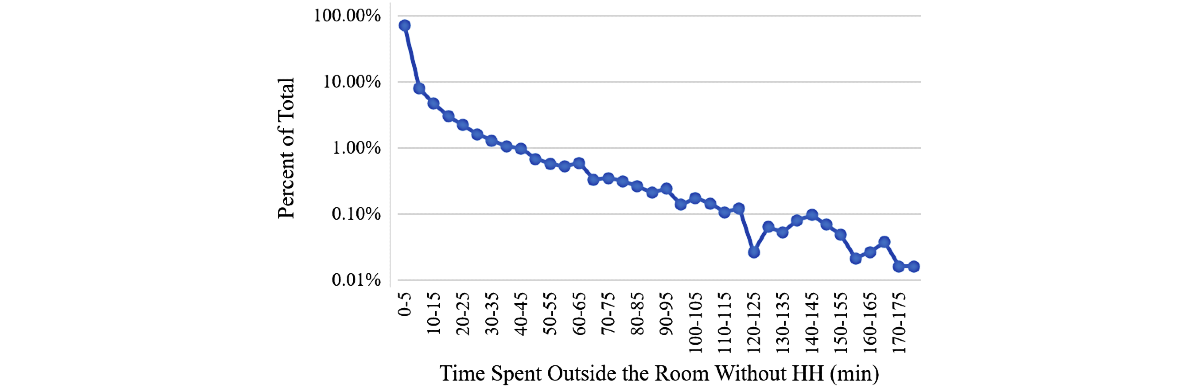
Distribution of time between previous hand hygiene and entrance to a room. HH: hand hygiene; min: minutes.

Clack et al [27] reported that, on average, 14.2 hand-to-surface exposures per minute happened in an intensive care unit, where 46% were outside the patient zone. This indicates that the time duration spent outside the patient room, including visiting the previous patients or staying in the hallways, can play an important role in infection transmission since this will give the HCWs enough time to contact the environment. Figure 3 shows the time duration spent outside the patients’ rooms before entering a specific room without HH action.

It was observed that more than 35% (2449/6887) of the nurses spent less than 5 minutes outside the patients’ rooms with no handwashing action. In addition, about 75% (5091/6887) of the nurses spent 0-25 minutes outside the rooms without performing HH. It is likely that the conventional performance metric, compliance rate, is unable to accurately estimate the patient’s exposure risk to an infection without considering all these factors.

### Formal Definition

**Figure 4:**
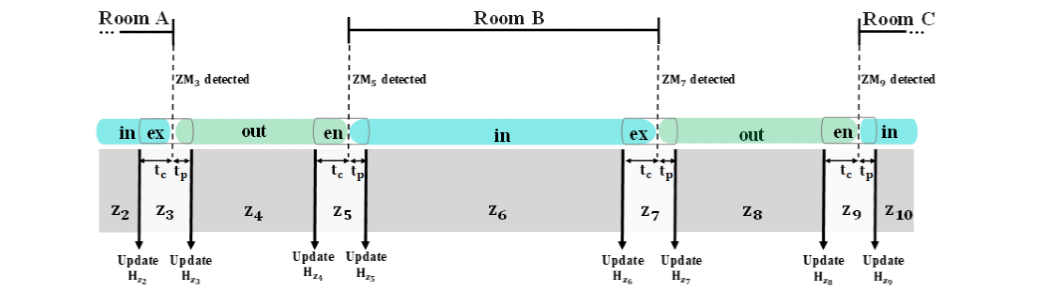
Zones and timestamps used in calculating the patient exposure risk. in: inside; ex: exit; en: entrance; z: zone; H: History; ZM: zone marker.

The patient exposure risk is defined considering 4 zones shown as *z_i_* (Figure 4), including outside the room (“out”), inside the room (“in”), room entrance (“en”), and room exit (“ex”). The “en” or “ex” zones (*z_i_* with *i=2k+1*, where *k* ∈ *W*) are specified by 2 time constants: *t_c_* and *t_p_*.

An HH opportunity is compliant if HCWs wash their hands within *t_c_* seconds before and *t_p_* seconds after entering or exiting the room detected by zone markers (*ZM_i_*) (Figure 4). *t_c_* is defined for the scenarios where the first and the fourth moments of HH are combined. For instance, if the HCW performs HH upon exiting a room (moment 4) and immediately enters another room (moment 1) without touching other surfaces, moment 1 and moment 4 are combined, and one handwashing action is sufficient in this scenario. Therefore, the missed HH opportunities that occur within *t_c_* seconds after the last HH action are not counted. HCWs can wash their hands after *t_p_* seconds of their entrance if they forget to do so. This parameter can also be used to issue a reminder for missed HH opportunities.

The patient exposure risk, denoted by PER, for room *x* is calculated as follows:




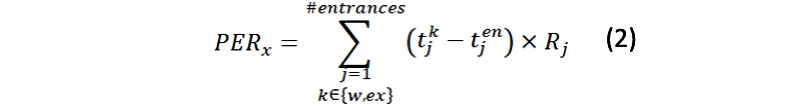




where *t* denotes time and *w* is a binary representation of handwashing action. *w=1* and *w=0* show that handwashing is performed and missed, respectively. *t_j_^k^ – t_j_^en^* is the time duration spent in room *x* by *j^th^* entrance without HH. Two scenarios (*k ∈ {w, ex}*) are modeled in Equation 2. The first scenario is when the caregiver washes their hands inside the room after *t_j_^w^* seconds (*t_j_^w^ – t_j_^en^*), and the second scenario is when they do not wash their hands until they exit the room. In this case, the total time that they spent in this room is considered as (*t_j_^ex^ – t_j_^en^*). The parameter *R_n_* (Equation 3) represents the risk associated with each HCW entering room *x* and is calculated as follows:




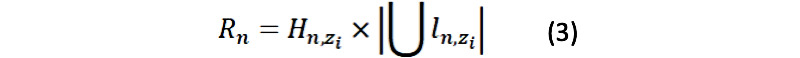




*H_n,z_i__* denotes the history of *n^th^* staff entering *i^th^* zone. This variable represents the risk of infection that staff ‘*n*’ accumulates over time before entering *z_i_*. *l_n,z_i__* is a set of rooms visited by *n^th^* staff without performing HH before entering *z_i_*. Therefore, |∪ *l_n,z_i__*| calculates the number of unique rooms visited by this staff without any handwashing action before entering *z_i_*. For the “en” or “ex” zones, where *i=2k+1*, the *H_n,z_i__* is calculated as Equation 4, and for the “in” or “out” zones, where *i=2k*, the *H_n,z_i__* is calculated as Equation 5.




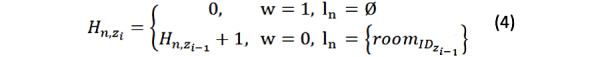







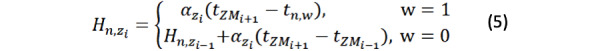




*α_z_i__* and *ZM_i_* are the risk factor and zone marker for zone *i*. We assume that each zone might have different risk factors based on the infectious agents that might be present in each individual zone (*α_z_i__*). For example, the risk factor for the isolation room will be different from that of regular patient rooms, or the risk factor for the hallway will be different from that of patient rooms. In the “en” or “ex” zones, the history of HCW will be increased if they do not perform any handwashing action (*w=0*) upon entering or exiting the room (moment 1 and moment 4). In the “in” or “out” zones, where *i=2k*, we incorporated the time duration that the HCWs did not wash their hands. There are 2 cases that are modeled in Equation 5. The first case is that the caregiver washes their hands inside the zone after *t_n,w_* seconds (*t_ZM_i+1__ – t_n,w_*), and the second case is when they do not wash their hands until they exit the zone. In this case, the total time that they spent in this zone (*t_ZM_i+1__ – t_ZM_i–1__*) is calculated and added to their current history (*H_n,z_i–1__*). The history (*H_n,z_i__*) resets whenever handwashing action is performed (*w=1*)

## Results

**Figure 5:**
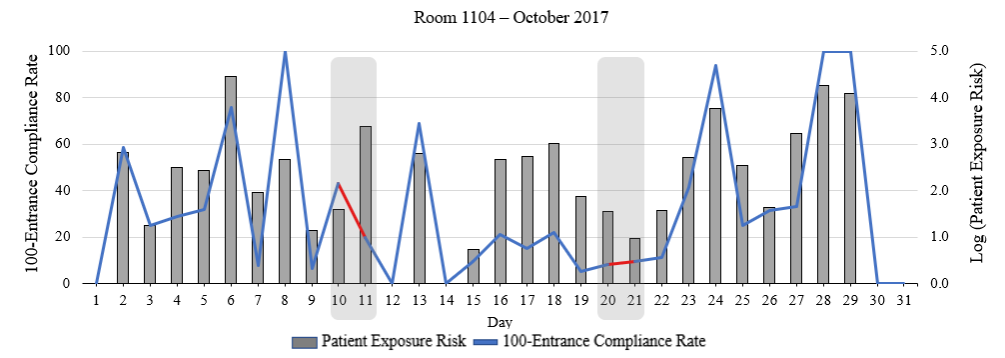
Entrance compliance rate (ECR) and patient exposure risk for room 1104 in October 2017.

In this section, we compared the ECR and the proposed patient exposure risk with risk factors set to *α_z_i__=1*. Since the ECR is calculated based on the number of HH actions performed upon room entrance, this parameter is already included in our patient exposure risk. Therefore, we expect to see some correlations between patient exposure risk and (*100 – ECR*). However, the critical points are where these 2 parameters are not in agreement; this indicates that other factors may play an important role in the prediction model, which were ignored in the ECR. As an example, Figure 5 provides a comparison between patient exposure risk and (*100 – ECR*) for room 1104, in October 2017. Although the correlation coefficient between these 2 variables was *r*=0.83 (*P*<.001), there are 7 points where the 2 parameters provided conflicting results. For example, considering October 10 and 11, the ECR values on these 2 days showed that the HH performance was significantly higher on October 11 versus October 10, whereas the analysis of patient exposure risk indicated that the patient was likely exposed to a substantially greater risk of infection on October 11 compared with October 10.

On October 10, 3 out of 7 entrances to Room 1104 had a chain of missed HH opportunities. As summarized in Table 2, these 3 HCWs visited a total of 5 rooms, missed 6 HH opportunities, and spent about 16.45 minutes outside the room without washing their hands before entering Room 1104. However, in total, they all spent only 1 minute in the patient’s room. Thus, the overall patient exposure risk for Room 1104 was as low as 38. On this day, 57% (4 out of 7) entrances into this room were compliant with HH.

**Table 2:** An example to compare the patient exposure risk and entrance compliance rate (ECR).

Days	#ent^a^	#ent with H^b^>1	|∪ *l_n,z_i__*|	#missed HH^c^	Time outside the room (minutes)	Time inside the room (minutes)	Patient exposure risk	100-ECR (%)
Oct 10	7	3	{1, 3, 1}	(1, 4, 1)	(5.68, 9.21, 1.55)	(0.03, 0.93, 0.05)	37.59	42.86
Oct 11	25	5	{1, 2, 2, 5, 2}	(2, 3, 3, 19, 5)	(20.2, 37.38, 53.05, 71.6, 13.92)	(3.6, 5.65, 1.16, 3.57, 2.95)	2388.24	20

^a^ent: entrance.

^b^H: History.

^c^HH: hand hygiene.

On October 11, there were 25 entrances into Room 1104, where 5 nurses did not wash their hands upon entering the room (ECR=80%). As summarized in Table 2, these 5 nurses visited 12 rooms, missed 32 HH opportunities, and spent almost 3.5 hours without washing their hands outside the room. In total, they spent about 17 minutes inside the patient room. This example obviously showed that the patient was in greater risk of becoming infected on October 11 compared with October 10 because these staff were more likely to carry infection. However, the ECR was approximately 25% higher on October 11, meaning that the performance of HH was better on this day. This example clearly explained that the traditional compliance rate only focuses on the adherence of HCWs to HH protocols and is not able to quantify and assess the potential patient exposure to infection. Similarly, October 20 and 21 showed another example in which ECR values were almost the same for both days, but the patient exposure risk was significantly different.

**Figure 6:**
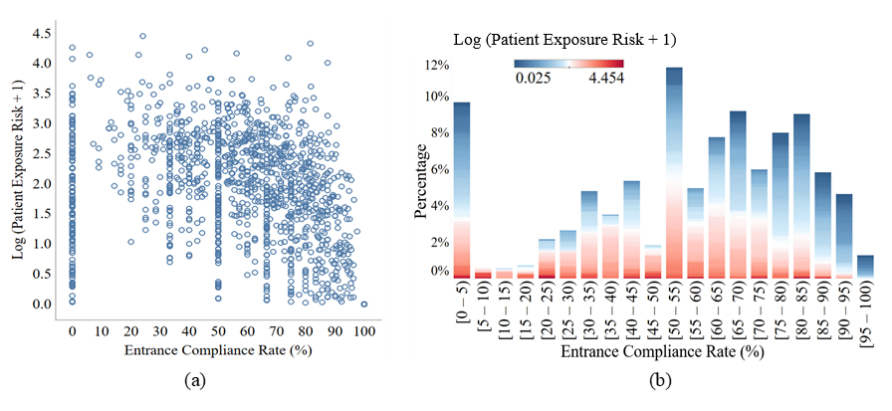
(a) Scatter plot of the log of the patient exposure risk and the entrance compliance rate (ECR); (b) distribution of the daily ECR for 20 rooms within 85 days (bars) and their corresponding patient exposure risk. The color change in this graph is proportional to log10 (patient exposure risk + 1).

Figure 6 (image A) depicts the reported daily ECR and its corresponding patient exposure risk for each room in the data set. There is no strong linear relation between these 2 parameters (*r*=0.7, *P*<.001).

The more the 2 parameters are uncorrelated, the better the role of factors incorporated in the patient exposure risk and excluded in the ECR are highlighted. Figure 6 (image B) shows the distribution of the daily ECR for all the days and rooms in the data set. The distribution of the patient exposure risk in each bar is illustrated using colors. Even in the columns that represent an acceptable range for ECR (eg, 90-95%) there exists a noticeable proportion of high patient exposure risk values (red areas). Moreover, the columns with a significantly low ECR (eg, 0-5%) can have values with low patient exposure risk (blue areas). This is due to the fact that patient exposure risk considers more than just missed HH opportunities upon entrance. For example, if all the nurses who enter a room, except 1 nurse, perform HH upon entrance, the ECR can demonstrate a high HH performance. However, if this nurse has a high “History”—has missed several HH opportunities, visited various rooms, or did not wash his hands for a long time—and spends a considerable amount of time inside the patient’s room, the patient may be exposed to a high risk of infection. On the other hand, if only one nurse enters a patient’s room and does not perform HH but spends only a few seconds inside that room, the ECR will be as low as 0 whereas the patient exposure risk is negligible.

## Discussion

### Principal Findings

The examples provided in this paper showed that the ECR cannot provide accurate information about the risk of contracting HAIs. On the other hand, patient exposure risk is designed to estimate the risk of infection for each individual patient. The patient exposure risk keeps track of consecutive missed HH opportunities and the risk accumulated by spending time inside and outside the patients’ rooms. It considers the source of infectious agents by identifying high-risk areas (eg, type of room and the medical condition of the patient inside the room). Areas such as soiled utility rooms, which are intended for decontamination, storage, and disposal of used equipment and waste, can be a potential source of infection. Moreover, the risk of transmission varies between different types of contagions present in the patient’s room [22,28]. Obviously, the ECR does not recognize the high risk caused by these areas whereas the patient exposure risk assigns a risk factor to each zone depending on its location and condition.

Several studies suggested that the risk of infection transmission is cumulative, yet health care settings rely on monitoring HH as individual binary events. In this paper, patient exposure risk is introduced to bridge the gap between the capabilities of new HH monitoring systems and infection prevention and control.

### Limitations and Future Work

While introducing this concept, we encountered different limitations such as a lack of prognostic data to validate the relationship between the patient exposure risk and infection rates. However, we plan to address these limitations in our future studies where we will include data from various hospital settings. We will present some examples of these limitations in the following section:

1. In this study, the patient exposure risk was estimated only for single-bed rooms since the current EMSs are not able to identify the exact location of the caregiver inside the room to detect the patient receiving care in multi-bed rooms. Incorporating localization inside the patient room is a possible future research area to overcome this limitation.
2. The proposed performance metric monitors the potential transmission of infectious agents through the HCWs’ hands by tracing their history of actions before entering each patient room. This is estimated using the number of consecutive missed HH opportunities, the number of unique rooms visited, and the time the HCWs spent without washing their hands before entering the patient’s room. In addition to these 3 parameters, the type of activities performed by the caregivers plays an important role in infection transmission. For instance, changing a patient’s dressing will potentially put the patient at greater risk of infection as opposed to assisting the patient with a wheelchair. The current EMSs are not able to identify the activities performed by the HCWs, and our data set does not contain this information. Therefore, the patient exposure risk concept will be extended in the future to include the types of activities performed by each staff, using artificial intelligence. This will be a new era of intelligent activity and behavior detection of frontline staff, which can help us identify the 4 moments of HH and the risk of exposure to infection accurately.
3. The analyzed dataset did not provide information regarding the patients in each room, and the patient exposure risk was not reported for each patient and was instead calculated for each patient’s room. However, this might lead to inaccuracies since a room can host different patients in a single day. Including this additional information, the patient exposure risk can be calculated for each patient without any changes in the formulation.
4. HH policies vary depending on the HCWs’ group assignment. For example, the staff responsible for pick-up and delivery of meal trays can go room to room without performing HH if there are no contacts with the patient or patient environment. In this paper, the patient exposure risk was only calculated for the nursing group who will need to perform HH in all the 4 moments of MOHLTC precautions. Future studies could explore the calculation of the patient exposure risk for other HCW groups according to their specific HH protocol. In addition, similar to the patient exposure risk that measures the risk of infection for the patients, a new concept will be introduced in the future to measure the risk of infection for HCWs who are at a high risk of infection.
5. Although in the patient exposure risk definition we considered the time duration that the HCWs spent in each patient’s rooms without handwashing action, it is also critical to include cases where the HH actions are performed but the contact time is over 15 minutes. For example, a close contact of more than 15 minutes is thought to increase the risk of infection transmission in the case of COVID-19 from staff to patient and vice versa [29]. Therefore, we will extend the patient exposure risk concept to a more general model that will incorporate such considerations.
6. Depending on the patient’s susceptibility, the number of microorganisms required to cause infection varies [22]. In other words, 2 patients can be exposed to the same number of pathogens but, depending on their immune status, the risk of developing an HAI might be different for them. This is another potential for future work to explore.
7. Although the patient exposure risk is introduced and tested in a rehab setting in this study, it can be easily extended to other environments and hospital settings. The integrated risk factors in the patient exposure risk formula can be customized depending on the hospital setting, the type of room, and the contagion in the room. By incorporating activity recognition into the system and accounting for patient’s susceptibility, we will be able to calculate the patient exposure risk tailored to each unit or hospital in the future. Currently, we are collecting data to test the patient exposure risk concept in different hospital environments.
8. Finally, in the future, we will investigate how to provide a standard rating system on patient exposure risk to enable the public to better understand the risk of infection exposure.

Several studies have demonstrated that performance feedback is effective in improving HH adherence [30-32]. Maintaining a positive culture is critical for sustaining improvements [33]. Criticizing staff by concentrating on the deviation of their compliance rate from a desired rate may result in a negative disciplinary tone. Infection prevention among patients is considered to be the most important reason among HCWs to perform HH [34]. We believe that by introducing patient exposure risk and reinforcing the need to decrease the risk of infection for both patients and HCWs [35], we will see further improvements in HH performance.

### Conclusion

Measuring the risk of HAIs for patients is an essential step for devising effective interventions for infection control. Controlling infection in health care settings requires continuous monitoring of HCWs’ handwashing behavior. HH behavior should be studied as a series of linked events in the chain of infection transmission. While conventional performance measures consider HH opportunities as binary events, the patient exposure risk enables us to evaluate the risk of missed HH opportunities based on time and location. As supported by different examples, the patient exposure risk helps predict the likelihood of patients becoming infected with HAIs. Future work will focus on providing a better estimation of the risk of contracting HAIs for patients by including additional factors such as activities performed by the staff in the room, estimated using sensors and AI. The same concept will be extended to estimate the HCWs’ exposure to infection risk.

## Abbreviations

ECR: entrance compliance rate
EMS: electronic monitoring system
HAI: health care-acquired infection
HCW: health care worker
HH: hand hygiene
MOHLTC: Ministry of Health and Long-Term Care
